# Supplementary material for: NK cells promote cardiac cell death and regulate myelopoiesis in myocardial infarction
Source: Nat Commun. 2026 Apr 1;17:4699. doi: 10.1038/s41467-026-71334-x (PMC13212716; doi:10.1038/s41467-026-71334-x)
Supplement: Supplementary file 1 — Supplementary Information [file 41467_2026_71334_MOESM1_ESM.pdf]

NK cells promote cardiac cell death and regulate myelopoiesis in myocardial infarction

First authors : Raphael Cohen & Vincent Duval

Last/Corresponding author : Hafid Ait-Oufella

Supplementary figures 1-25

Supplementary table 1

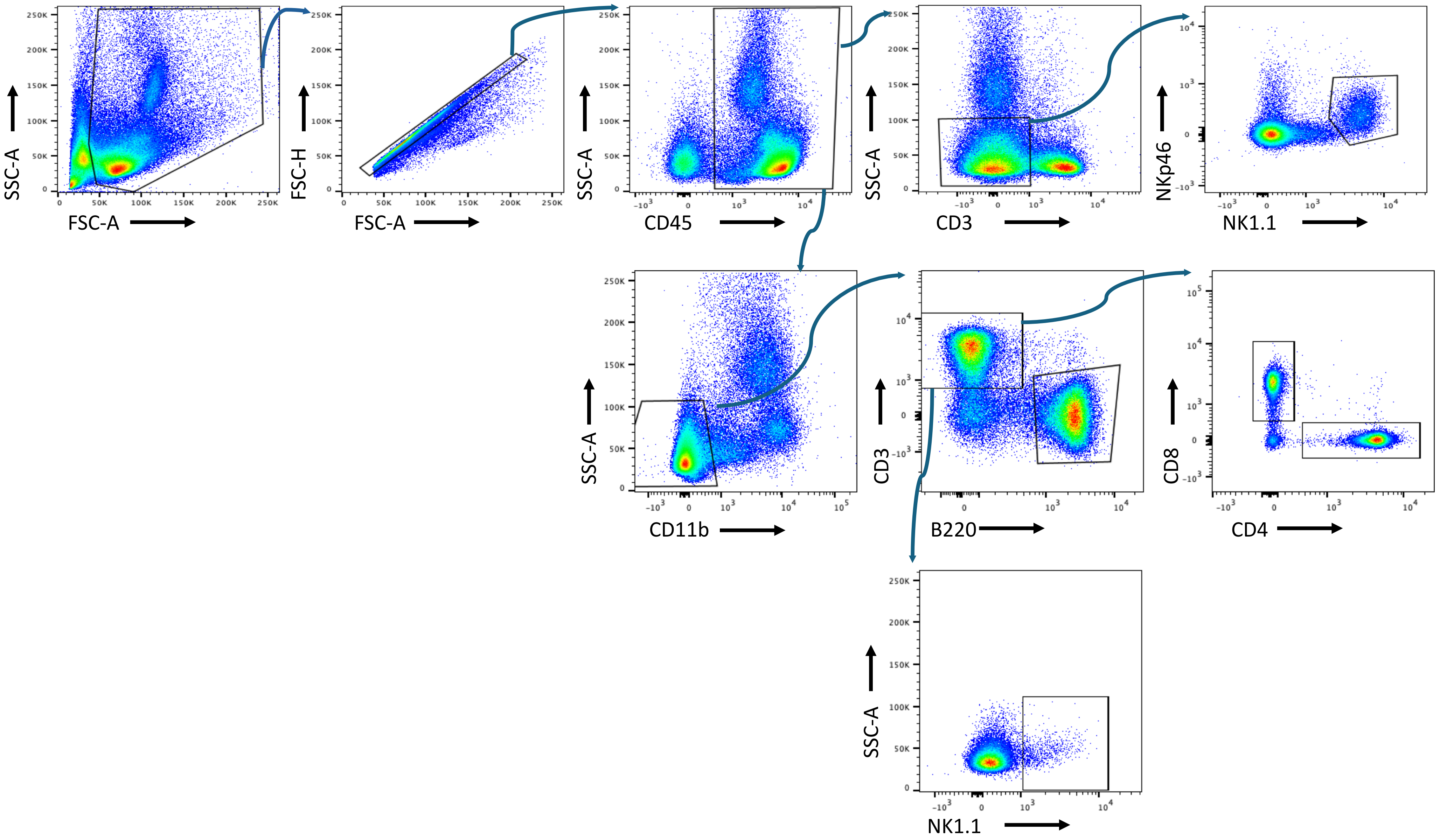

Supplementary Fig. 1. Gating strategy for non-myeloid cells (flow cytometry).

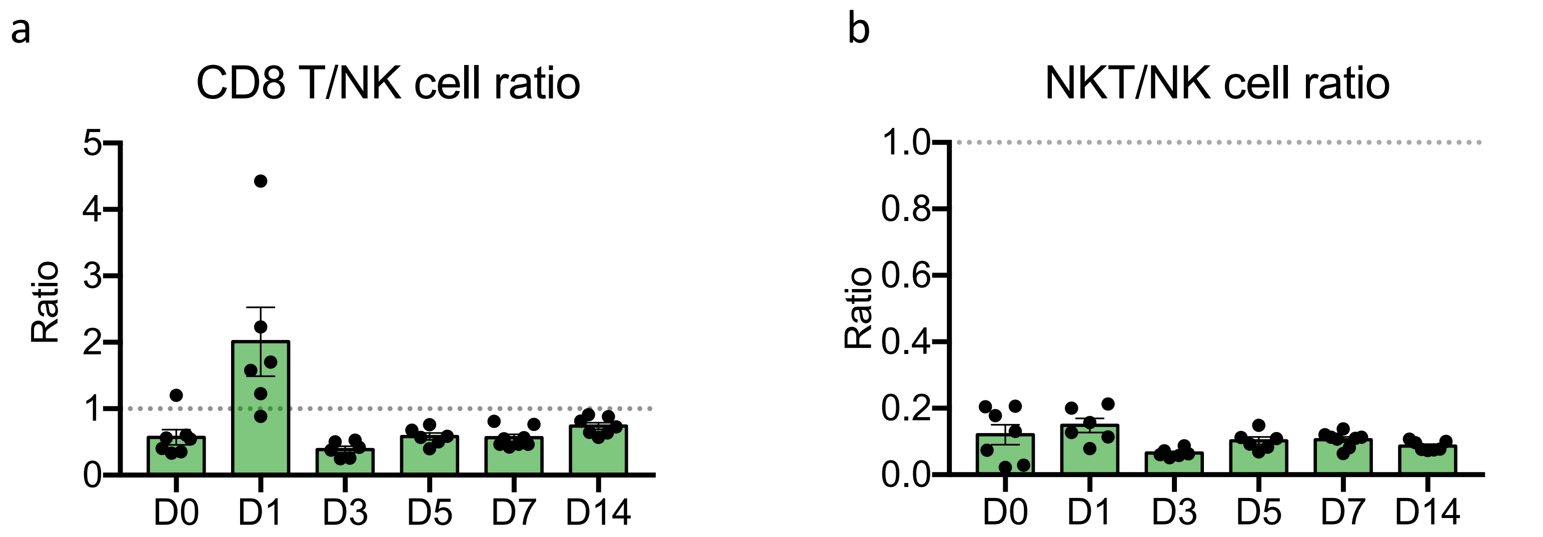

Supplementary Fig.2. Infiltrating populations in the ischemic heart tissue. Characterization of CD8/NK cell ratio (a) and NKT/NK cell ratio (b) in the ischemic heart time at different timepoints after MI, using flow cytometry. N=6/timepoint. Data are presented as individual, mean values +/- SEM.

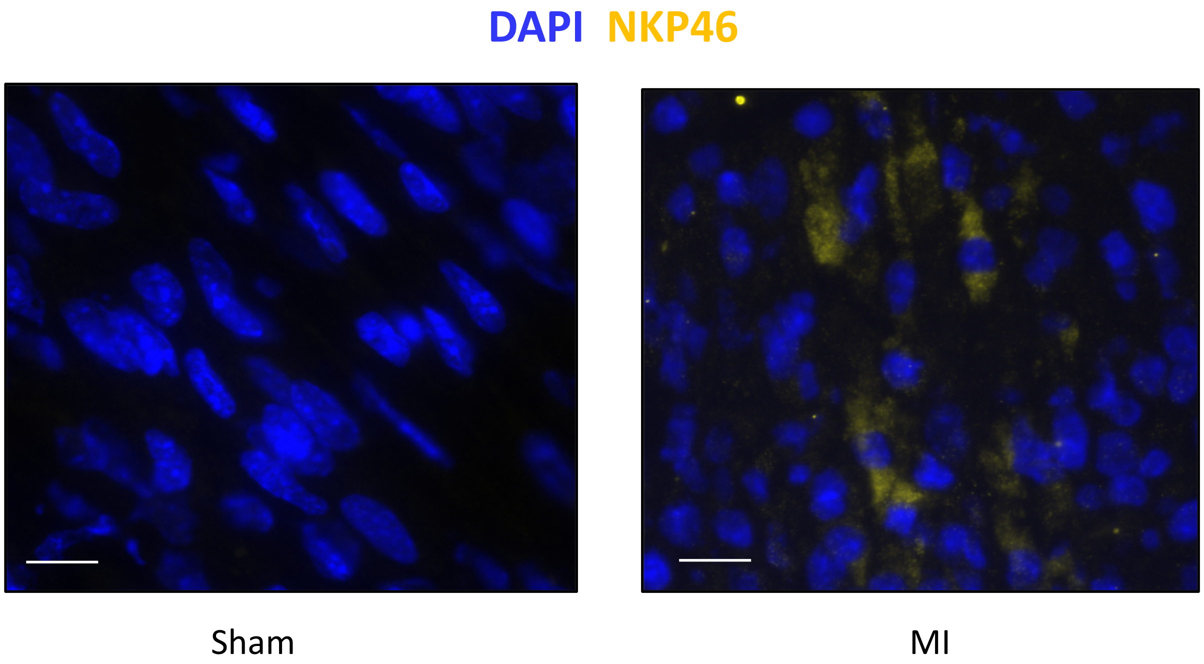

Supplementary Fig. 3. NK infiltration in ischemic heart tissue. Immunostainings in heart section at day 3 after Sham surgery or myocardial infarction (MI). NKp46+ NK cells were stained in yellow in the infarct area. Representative of 6 stainings/group. Scale bar 10 µm.

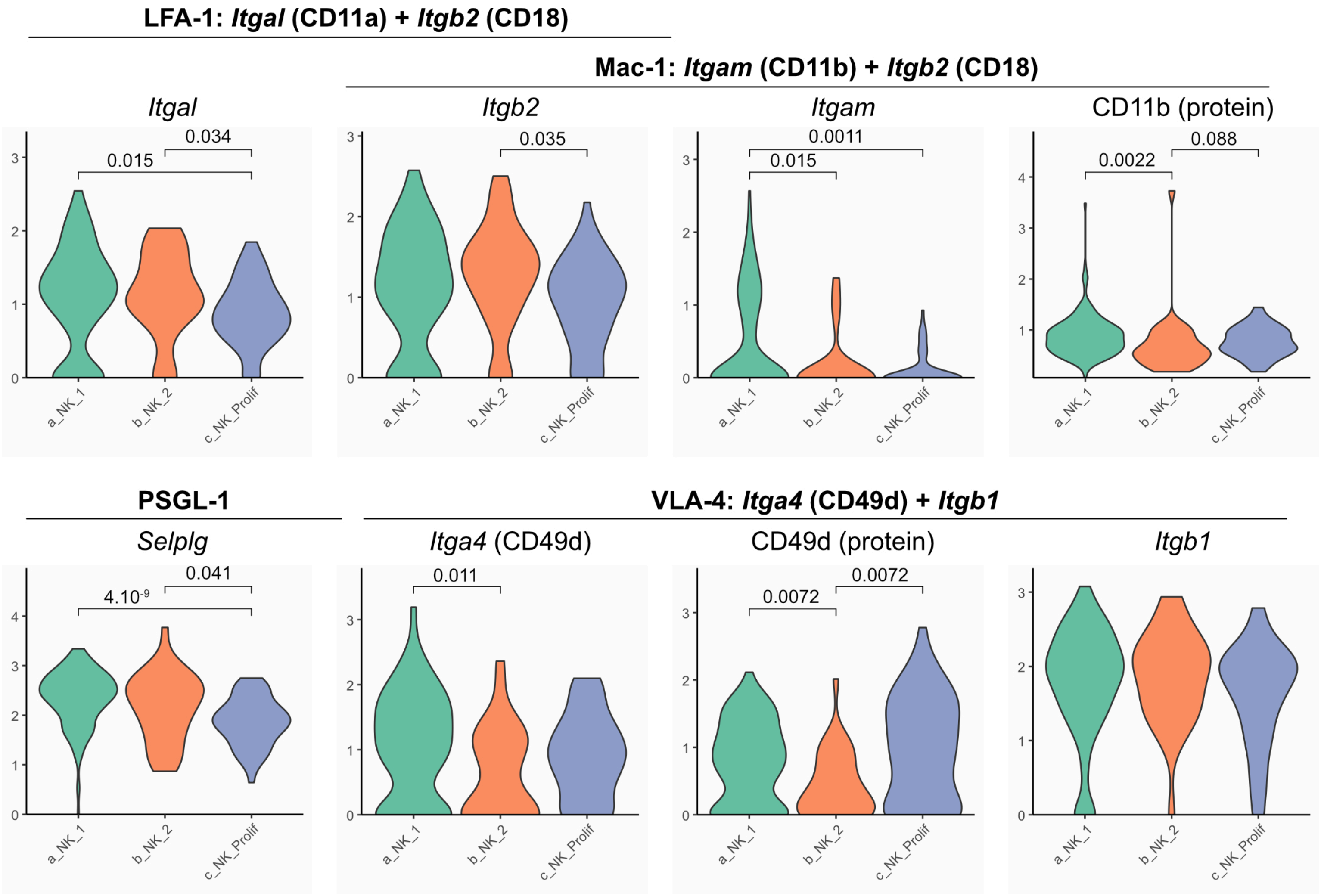

Supplementary Fig. 4. NK adhesion molecules. Gene expression levels of adhesion molecules in NK subsets identified by Sc-RNA seq at day 5 after MI. Statistical t test.

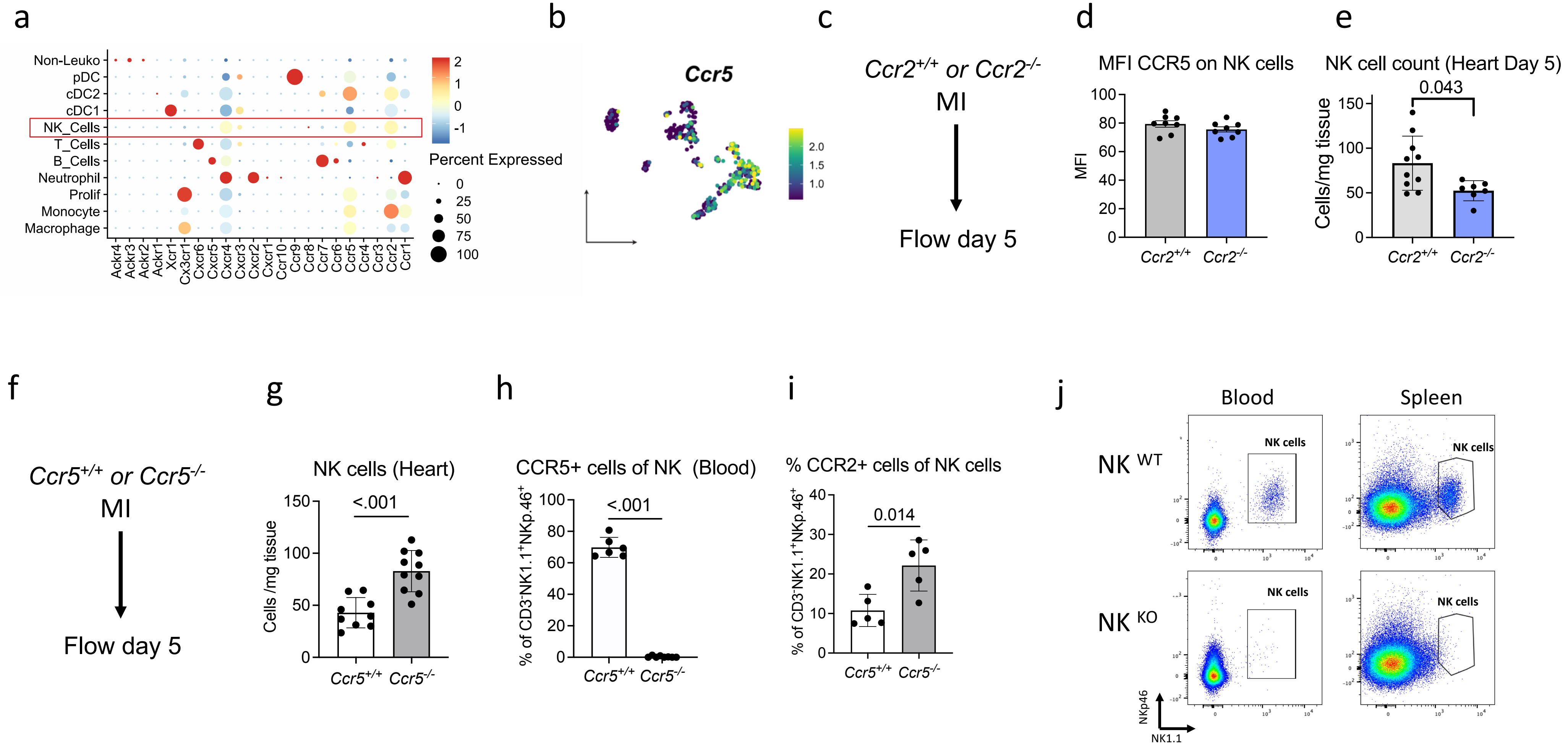

Supplementary Fig. 5. Chemokine pathways. a, expression of selected marker transcripts related to chemokine pathways in the leukocyte population within the heart tissue by Sc-RNAseq. b, expression of Ccr5 transcript in the populations in the heart at day 5 after MI. c, experimental protocol. d, CCR5 expression on NK cells (MFI) on *Ccr2*$^{+/+}$ and *Ccr2*$^{-/-}$ mice. e, NK cell infiltration in the heart in *Ccr2*$^{+/+}$ and *Ccr2*$^{-/-}$ mice at day 5 post-MI (N=10 *Ccr2*$^{+/+}$ and N= 7 *Ccr2*$^{-/-}$). f, experimental protocol. g, CD45+CD11b+NK1.1+Nkp46+ NK cell count at day 5 after MI in the heart of *Ccr5*$^{+/+}$ *(n=9)* and *Ccr5*$^{-/-}$ mice (n=10). h, validation of CCR5 deficiency using flow cytometry (n=6/group). i, CCR2 expression on circulating NK cells from *Ccr5*$^{+/+}$ and *Ccr5*$^{-/-}$ mice (n=5/group). j, Validation of NK deficiency in NK^{KO} mice using flow cytometry in the blood and the spleen. Data are presented as individual, mean values +/- SEM. P values were calculated using two-tailed Mann-Whitney test.

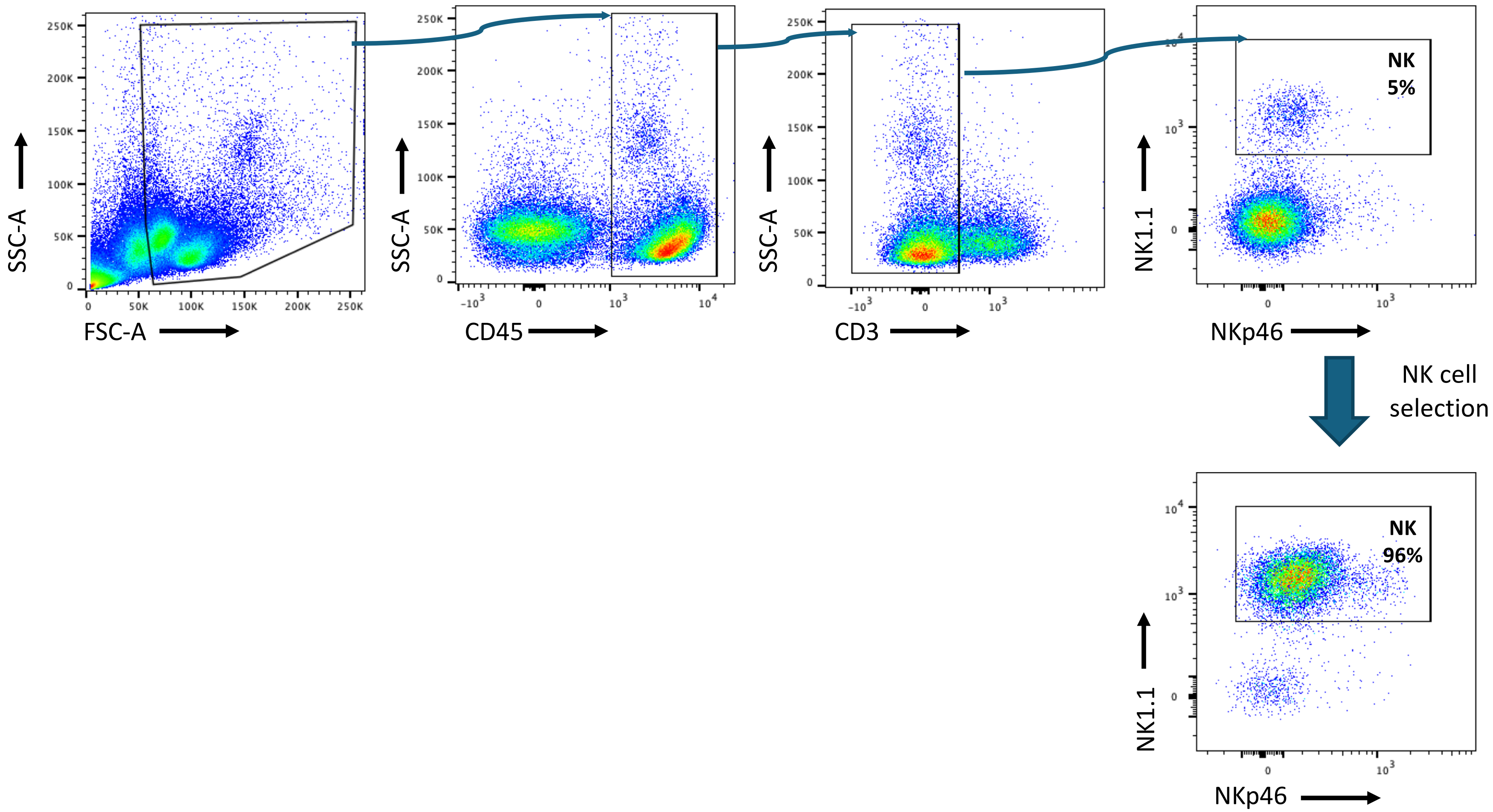

Supplementary Fig 6. NK cell isolation. Representative examples of NK cells before and after purification, using the NK cell isolation kit (Miltenyi Biotec) according to manufacturer's instructions.

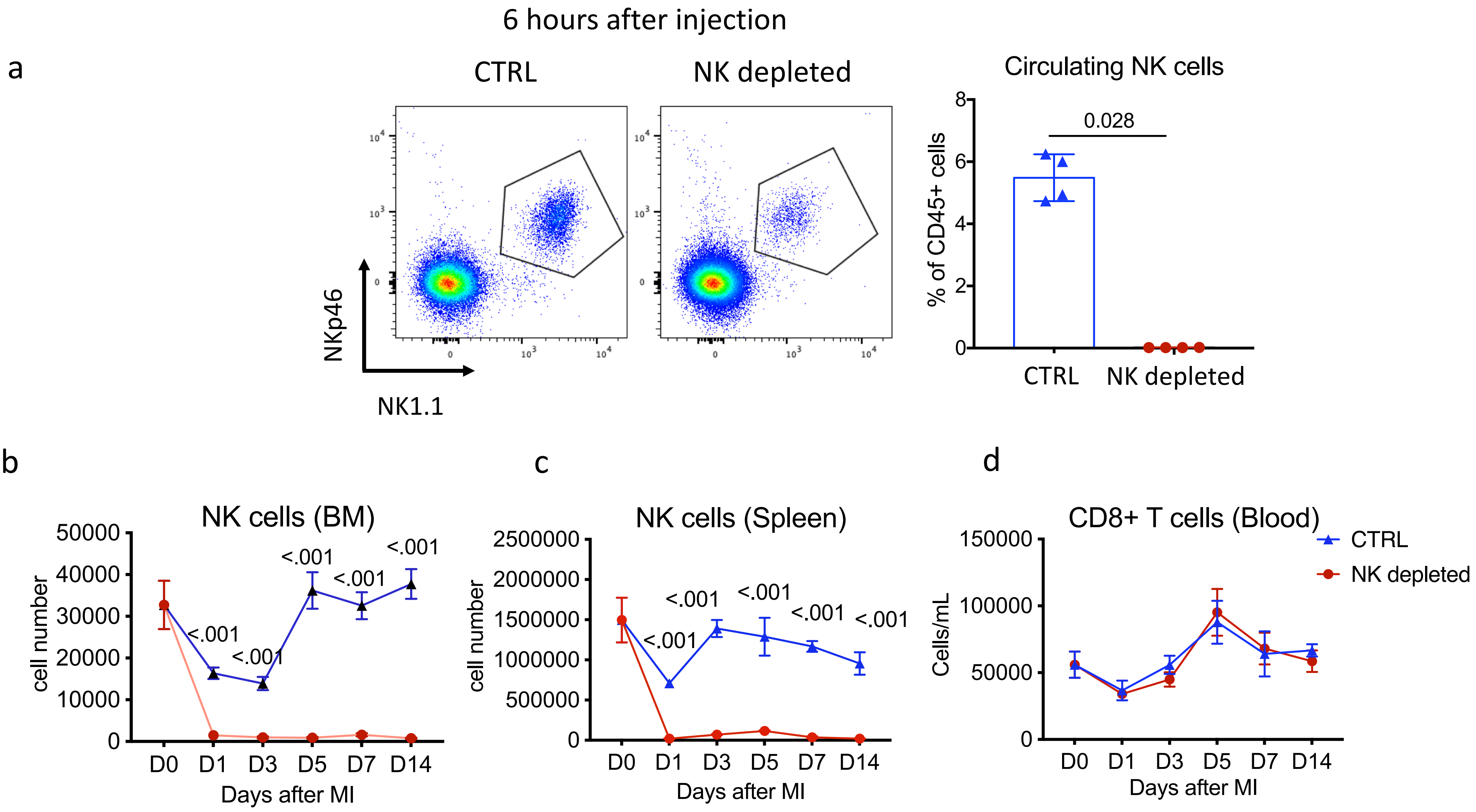

Supplementary Fig. 7. NK depletion following anti-NK1.1 mAb treatment. a, representative examples and quantitative analysis of NK cells in the blood 6 hours after injection of isotype control (blue) or NK1.1 mAb (red) (n=4/group). Kinetic of Nkp46+Nk1.1+ NK cell count in the bone marrow (b) and in the spleen (c) after isotype or Anti-NK1.1 mAb treatment in MI-operated C57Bl6 mice (N=6/group). D, Kinetic of CD8+ T cells in the blood in both groups. P values were calculated using two-tailed Mann-Whitney test (A) or Two-way ANOVA test (B, C, D). Data are presented as individual, mean values +/- SEM.

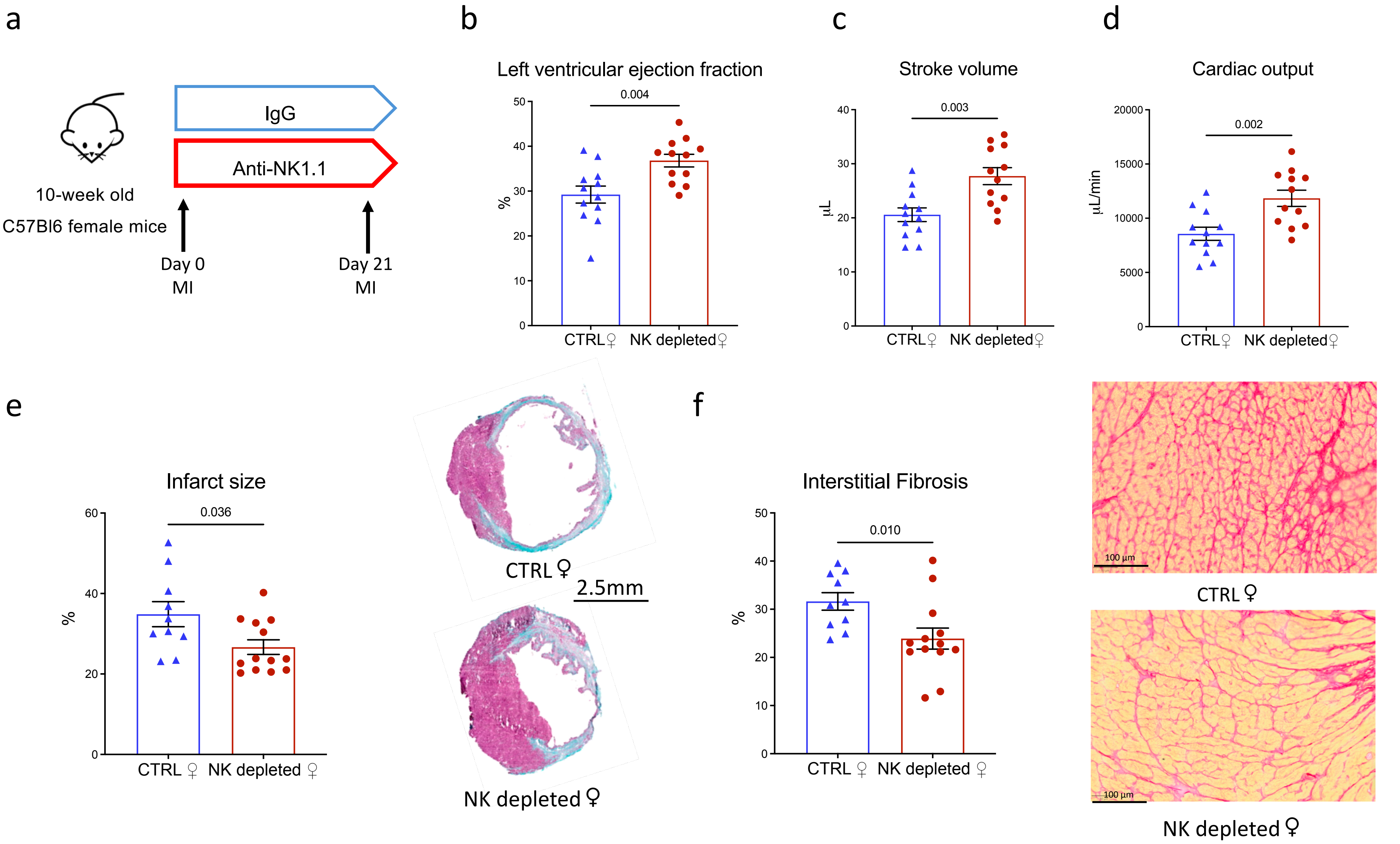

Supplementary Fig 8. NK depletion in female mice. a, experimental protocol. b, left ventricle ejection fraction (c) stroke volume and (d) cardiac output by echocardiography (CTRL blue, n=10 and NK depleted red n=13). e, representative photomicrographs and quantitative analysis of infarct size evaluation evaluated by Masson trichrome staining at day 21 (CTRL n=10 and NK depleted n=13). Scale bar 2.5 mm. f, representative photomicrographs and quantitative analysis of myocardial fibrosis evaluated by Sirius Red staining at day 21 (CTRL n=10 and NK depleted n=13). Scale bar 100 μm. P values were calculated using two-tailed Mann-Whitney test. Data are presented as individual, mean values +/- SEM.

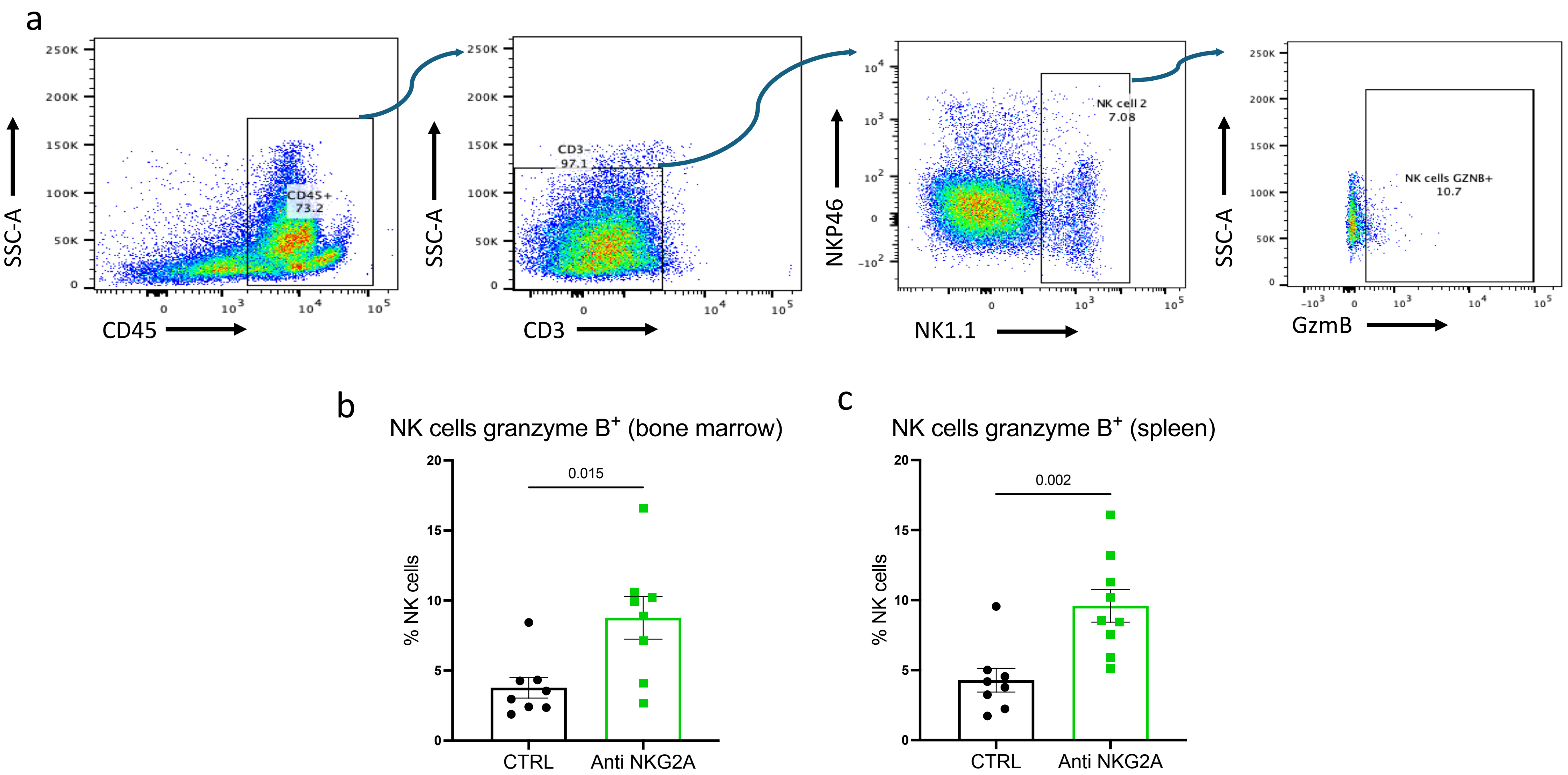

Supplementary Fig.9. Anti-NKG2A mAb induced NK activation. a, gating strategy to analyze Granzyme B content in NK cells. b, quantification of NK cells expressing granzyme B in the bone marrow. c, quantification of NK cells expressing granzyme B in the spleen (n=8 CTRL (isotype), n=9 Anti-NKG2A). Data are presented as individual, mean values +/- SEM. Two tailed Mann-Whitney test.

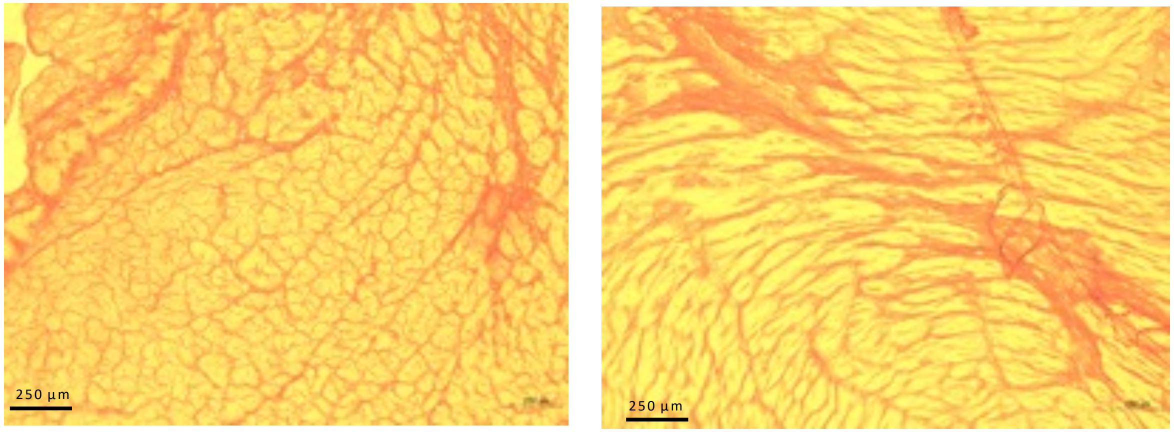

CTRL

Anti-NKG2A

Supplementary Fig.10. representative photomicrographs of myocardial fibrosis evaluated by Sirius Red staining, in the 2 groups of mice at day 21 (representative of 9 sections/group). Scale bar 250 µm.

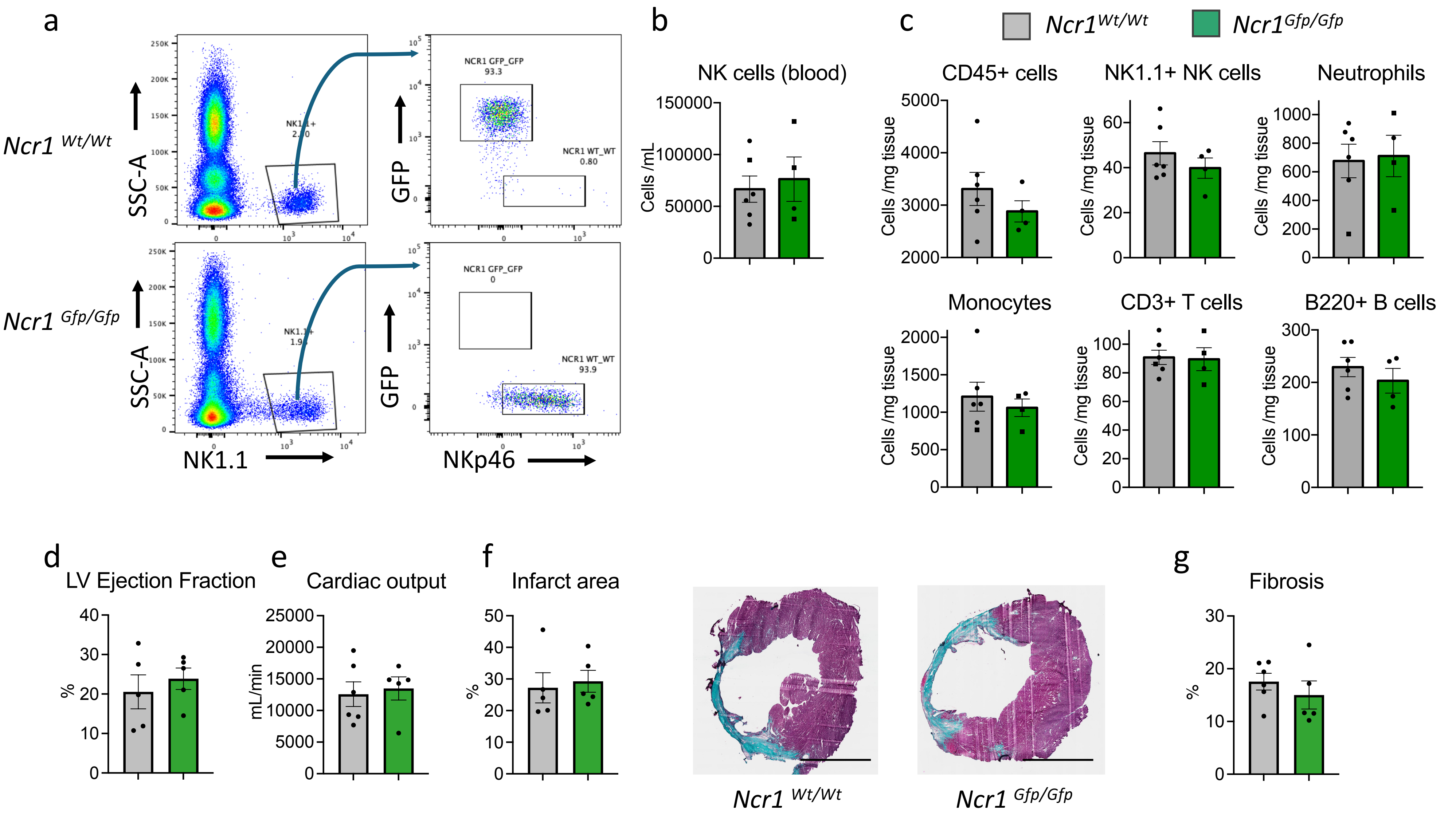

Supplementary Fig.11. Impact of Nkp46 receptor deletion on post-ischemic cardiac remodeling. a, gating strategy. b, CD45+CD3-NK1.1+ NK cell count in the blood. c, immune cell subsets in the ischemic heart tissue at day 5 after MI in *Ncr1*$^{Wt/Wt}$ control and Nkp46 deficient *Ncr1*$^{Gfp/Gfp}$ mice. d, e, echocardiography assessment at day 21 after MI. f, infarct size quantification and representative pictures at day 21 (Masson Trichrome). G, fibrosis quantification at day 21 (Sirius Red). n=6 Control and n=5 Gfp/Gfp. Scale bar 2.5 mm. Data are presented as individual, mean values +/- SEM.

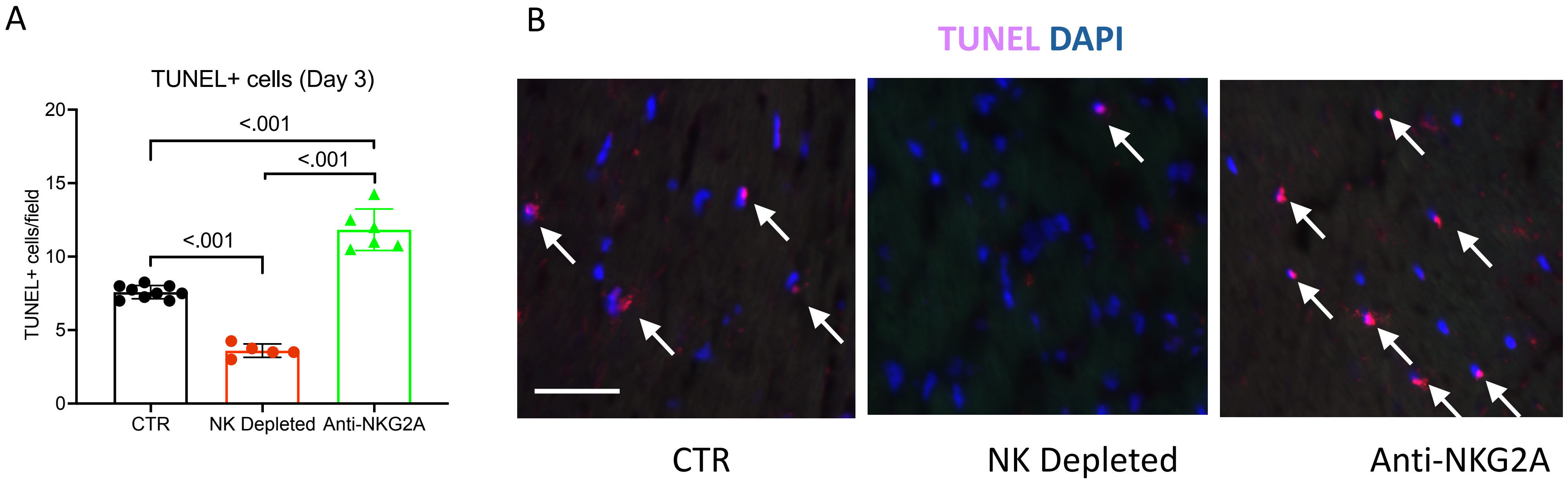

Supplementary Fig.12. NK cells promote cardiac cell death in the context of MI. A, quantification and **(B) r**epresentative examples of TUNEL+ cells (Red, white arrow) in the peri-infarct area of C57BL/6J mice treated with isotype control (N=9), depleting anti-NK1.1 mAb (N=5) or activating anti-NKG2A mAb (n=6/group). Data are presented as individual, mean values +/- SEM. Kruskall Wallis test. Scale bar 20 μm.

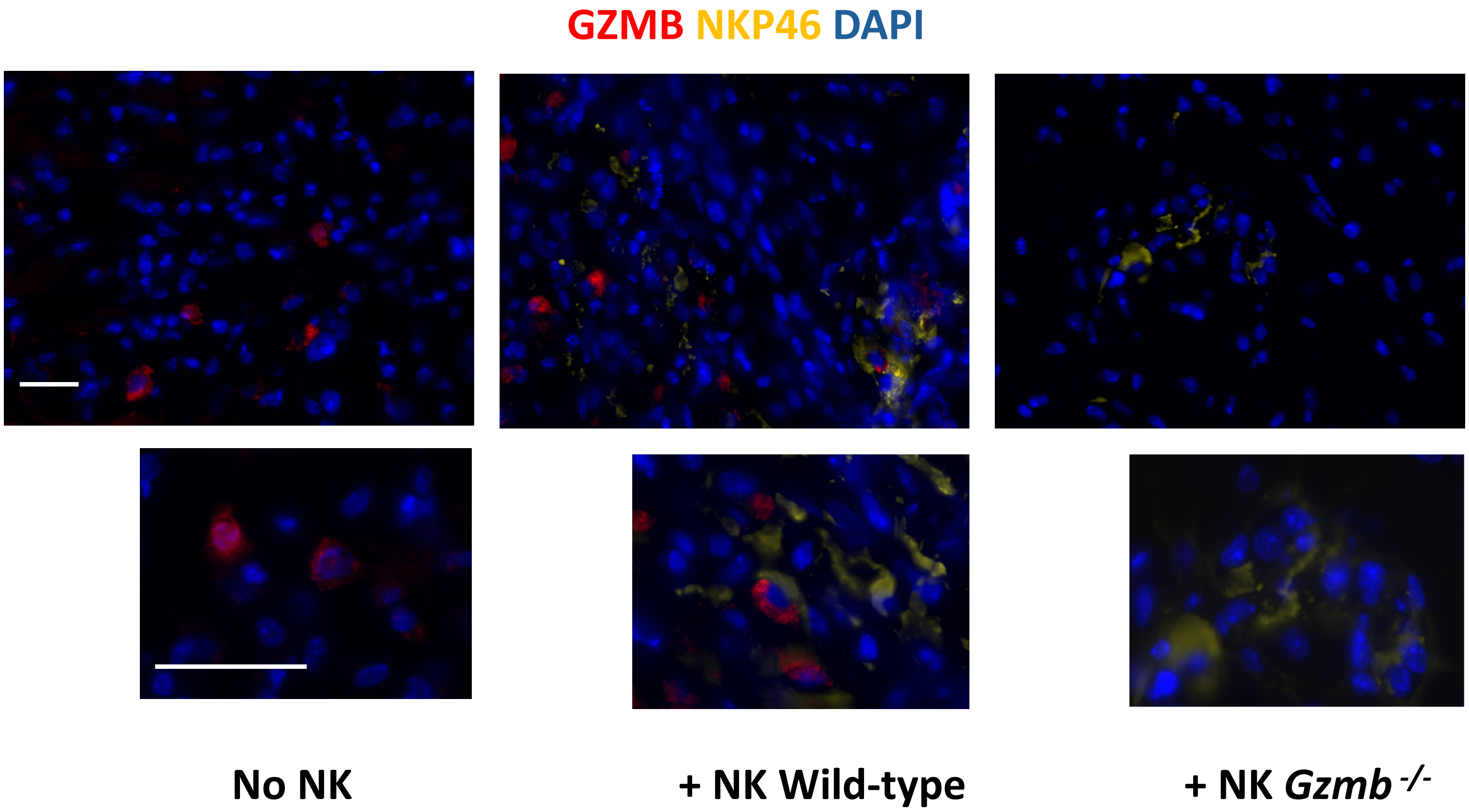

Supplementary Fig 13. NK cell repopulation. Immunostainings at day 3 after MI in the ischemic heart tissue of NK^{KO} mice supplemented or not with purified Wild-type or *Gzmb*$^{-/-}$ NK cells (representative of n=3/ group). Scale bar 50 µm.

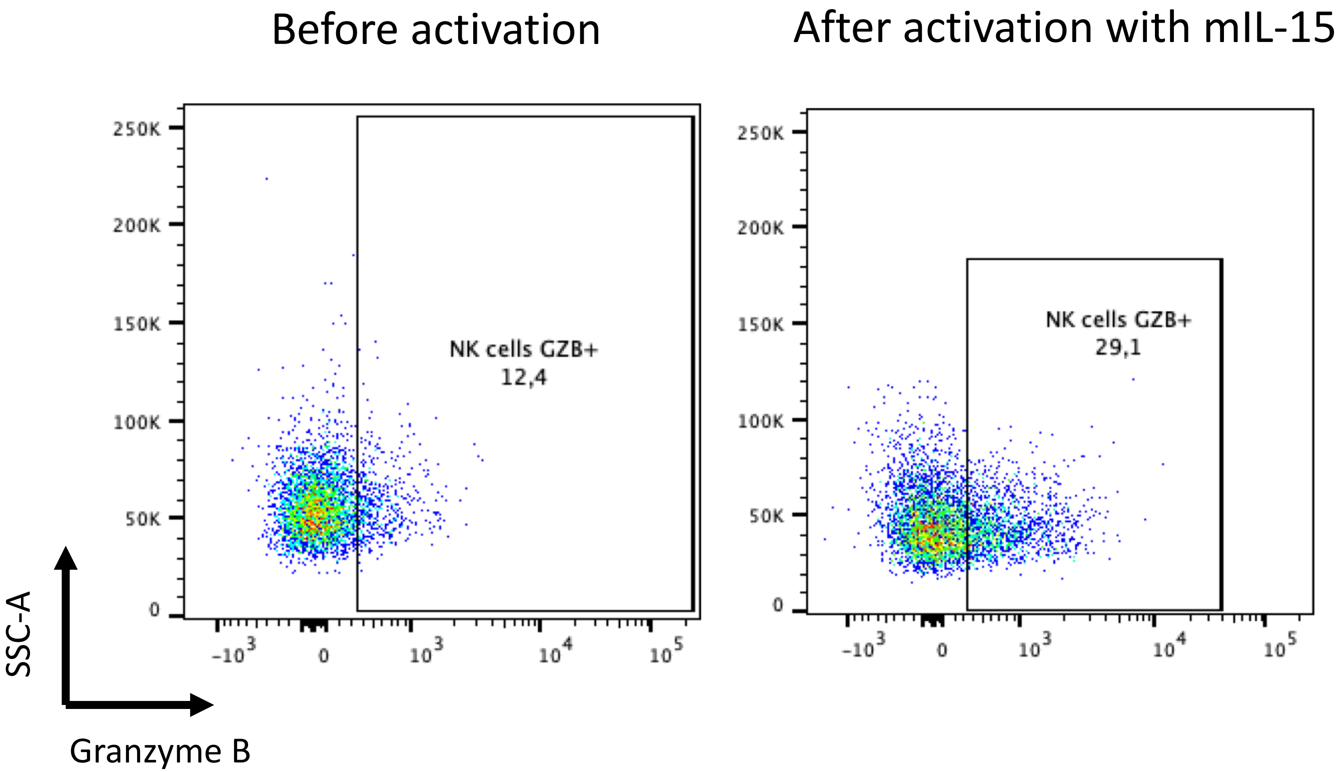

Supplementary Fig 14. NK cell activation ex vivo using mIL-15 using flow cytometry with intracellular staining for Granzyme B. NK cells were defined as CD3-Nkp46+NK1.1+ cells.

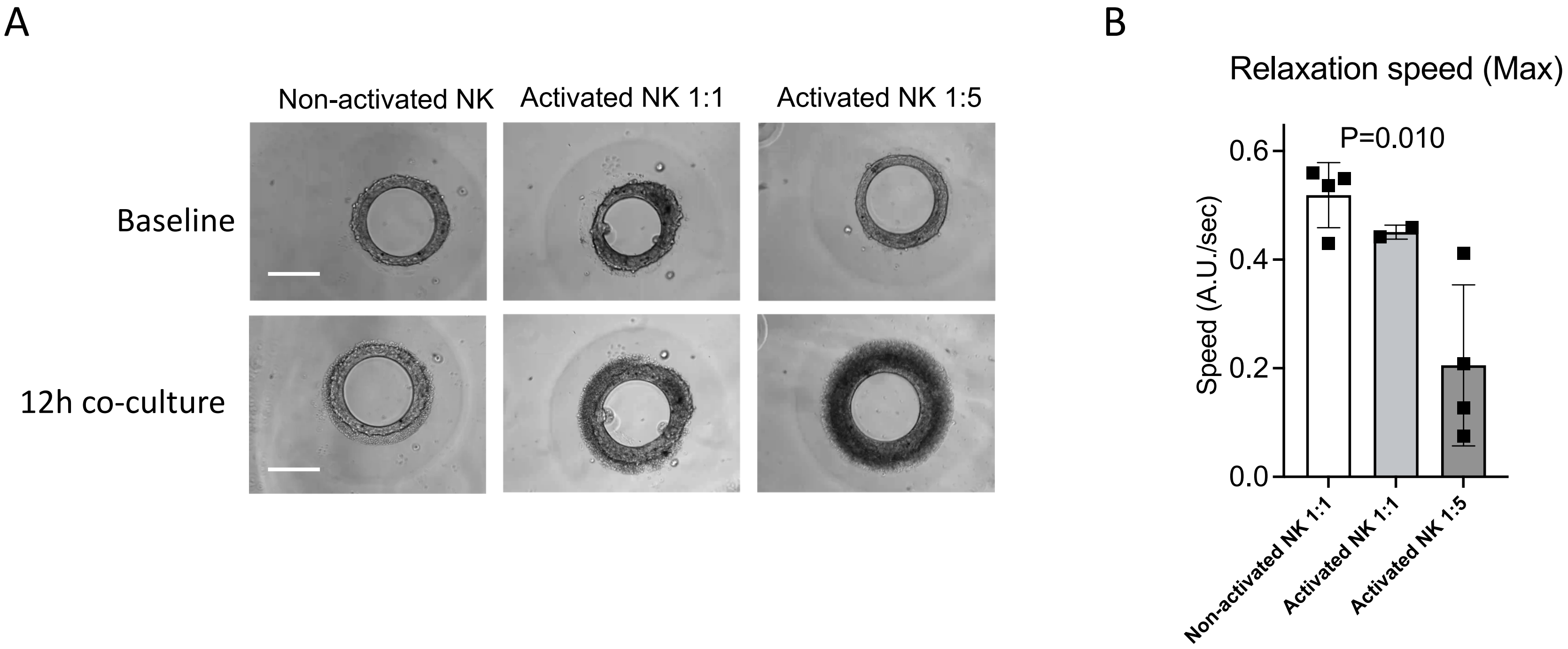

Supplementary Fig 15. co-culture between human 3D cardiac rings and NK cells. A, representative pictures of cardiac organoids before and after 12 hours of co-culture with NK cells. B, maximal relaxation speed after 12 hours of co-culture with non activated NK cells 1:1 ratio (N=4), activated NK cells 1:1 ratio (N=2) and activated NK cells1:5 ratio (N=4). Data are presented as individual, mean values +/- SEM. ANOVA test. Scale bar 1 mm.

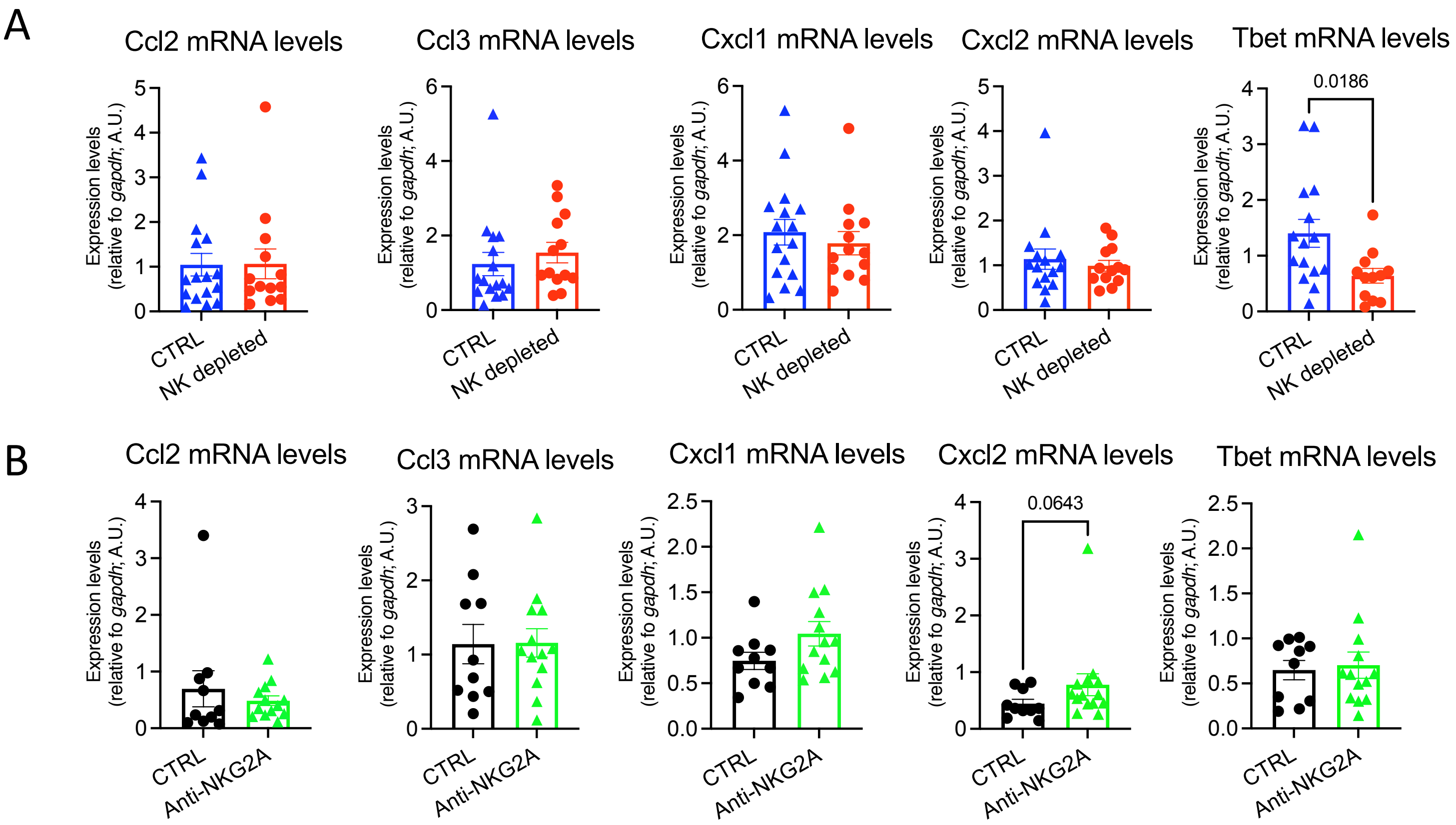

Supplementary Fig 16. **Impact of NK modulation on immune profile in the ischemic heart.** Myocardial infarction was induced on Male C57Bl6 males and mRNA levels were quantified in the heart at day 7. A, mRNA levels in mice treated with isotype control (CTRL, N=15) or depleting anti-NK1.1 mAb (N=13). B, mRNA levels in mice treated with isotype control (CTRL, N=10) or activating anti-NKG2D mAb (N=14). Data are presented as individual, mean values +/- SEM. Two tailed Mann-Whitney test.

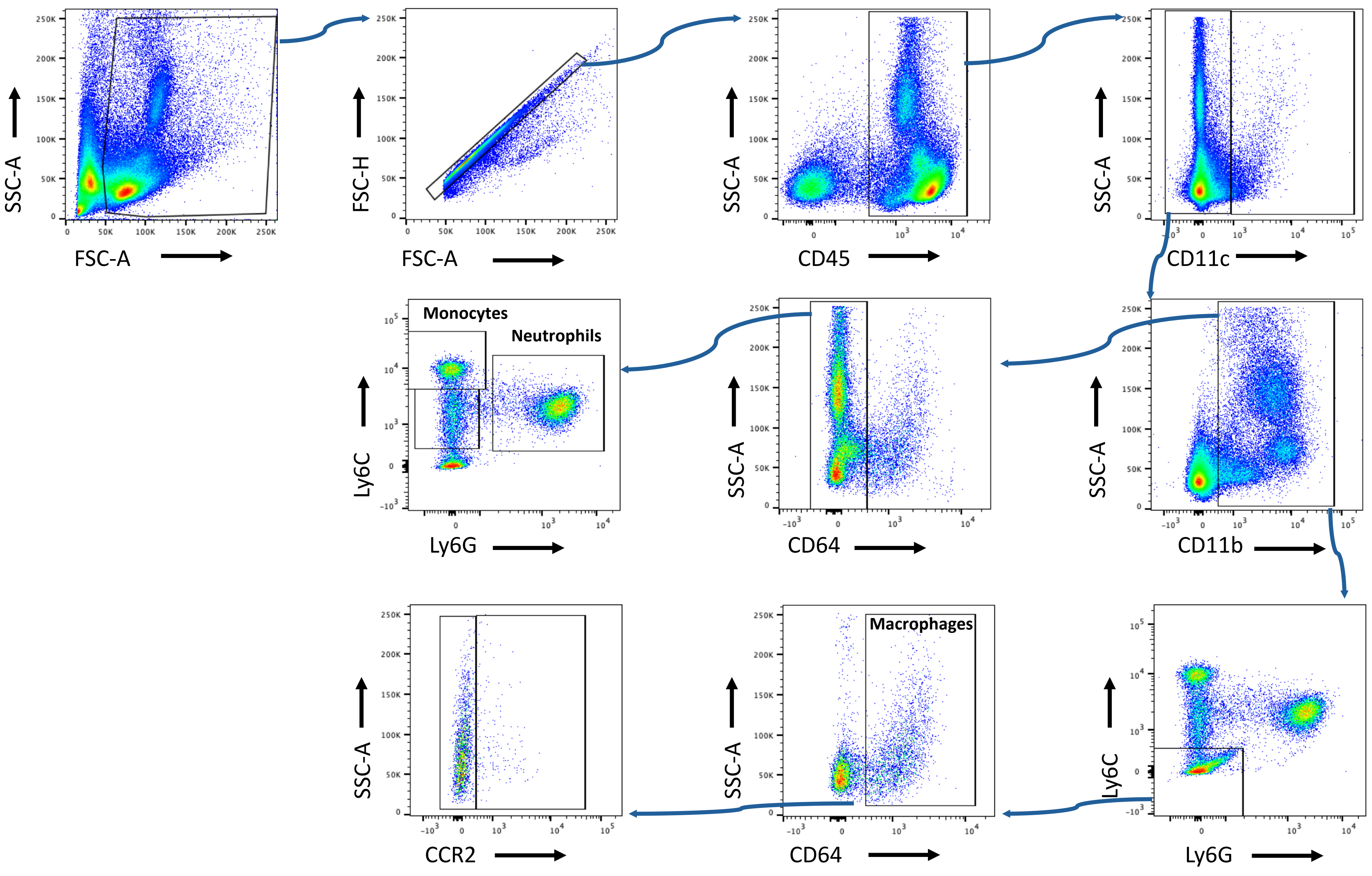

Supplementary Fig 17.gating strategy for myeloid cells (flow cytometry).

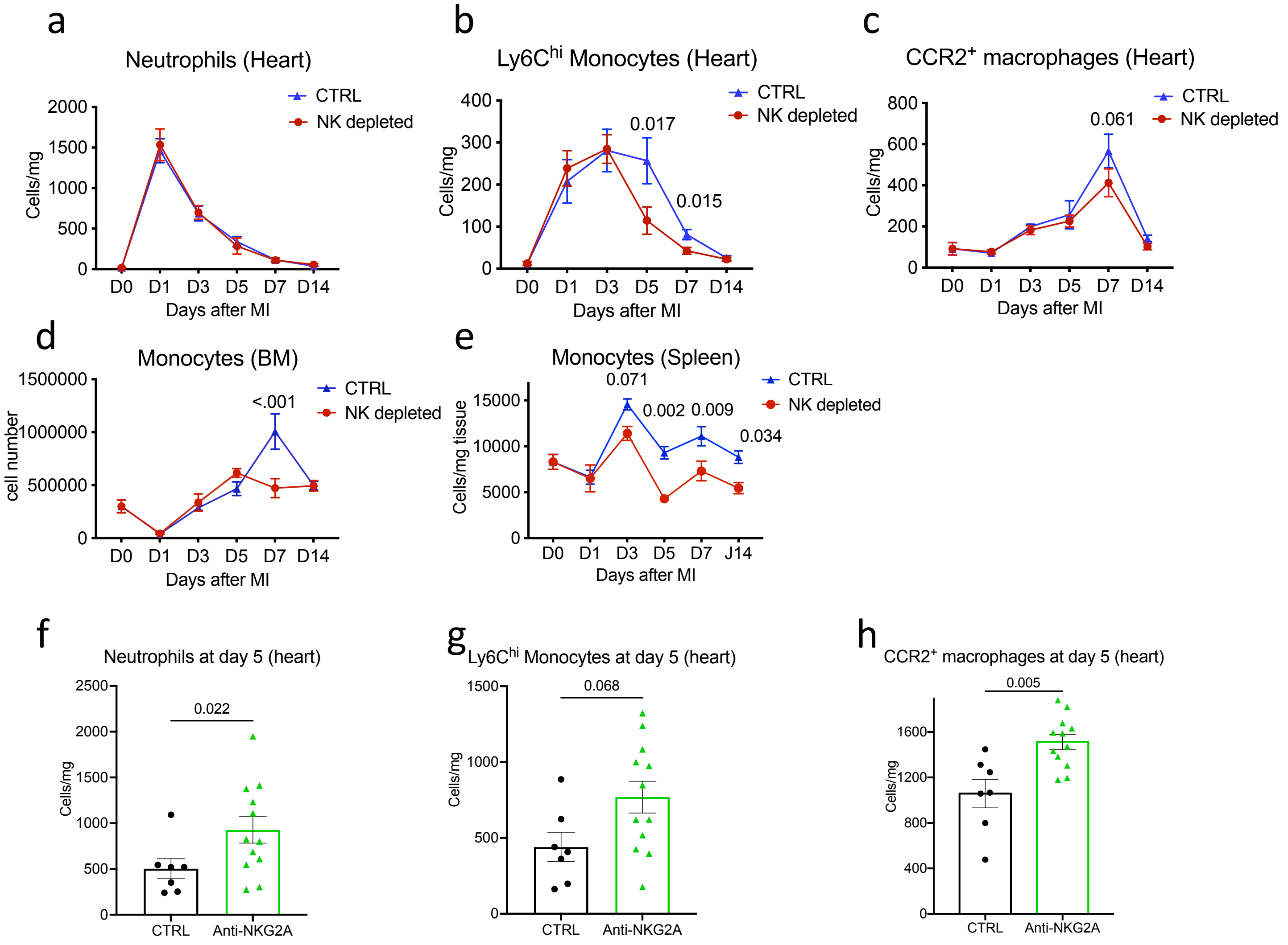

Supplementary Fig 18. Impact of NK cell modulation on myeloid cell trafficking. Kinetic of neutrophils (a), Ly6cHigh monocytes (b) and CCR2+macrophages (c) in the ischemic after MI in CTRL and NK depleted mice. Kinetic of monocytes in the bone marrow (d) and in the spleen (e) following MI in CTRL or NK depleted mice. CTRL (blue, n=7/5/6/6/8/7 respectively at day 0/1/3/5/7/14) and NK depleted mice (red, n=7/6/7/6/8/7 respectively at day 0/1/3/5/7/14). Neutrophils (f), Ly6cHigh monocytes (g) and CCR2+macrophages (h) in the ischemic heart at day 5 after MI in mice treated with isotype treated (CTRL) or anti-NKG2A treated mice (N=7CTRL and N=12 Anti-NKG2A). Data are presented as mean values +/- SEM. P values were calculated using two-tailed ANOVA test (a-e) and two-tailed Mann-Whitney test (f-h).

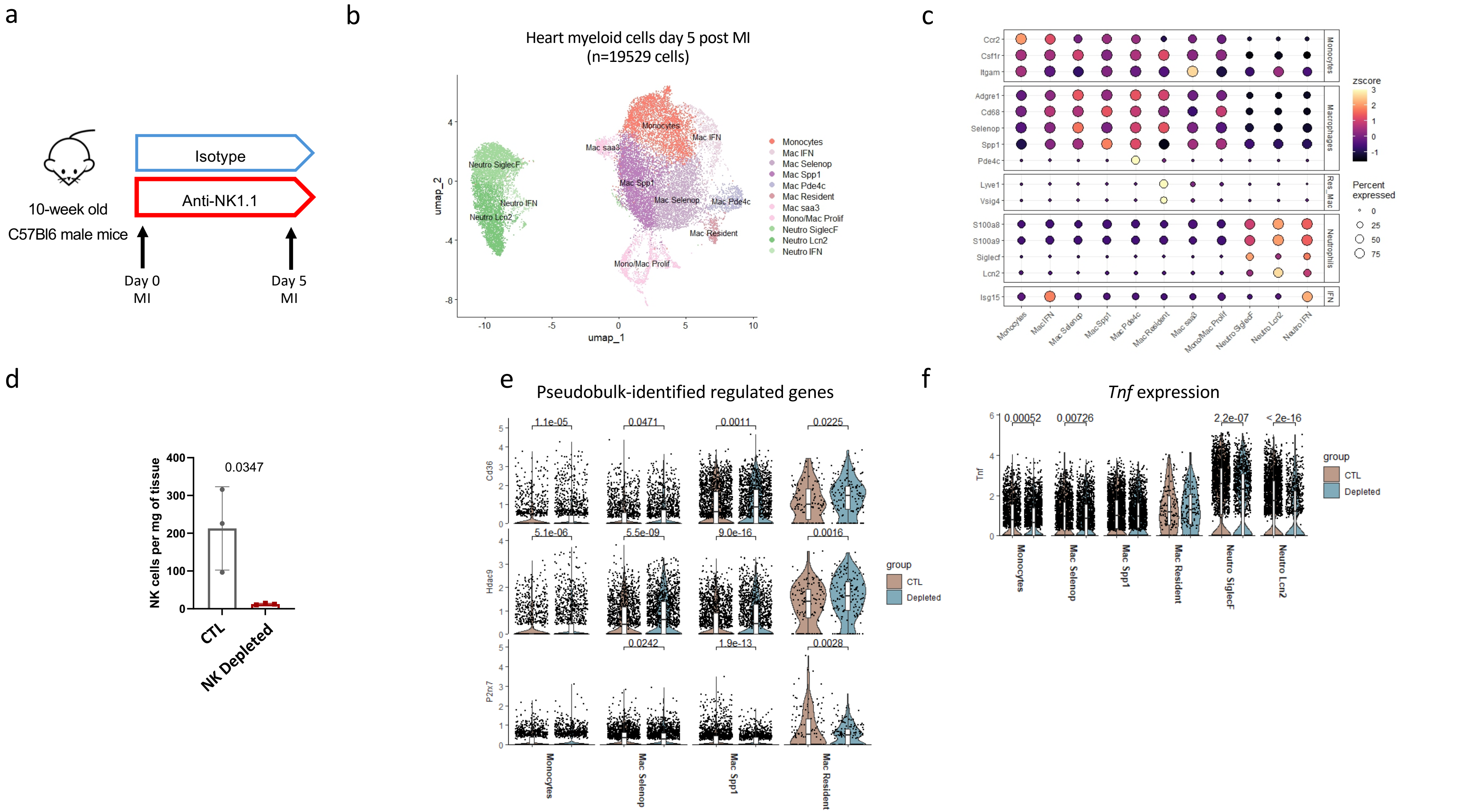

Supplementary Fig 19. **Impact of NK depletion on immune cell transcriptomic profile.** a: experimental protocol. B, Uniform manifold approximation and projection (UMAP) visualization of scRNA-seq profile of day 5 post MI heart myeloid cells. c, dot plot presenting the main markers used to determine cells subsets. d, number of NK cells per mg of total heart tissue. E, violin plot presenting *Cd36*, *Hdac9* and *P2rx7* gene expression in monocytes, Mac Selenop, Mac Spp1 and Mac resident in control and depleted groups; f: Violin plot presenting Tnf gene expression in monocytes, Mac Selenop, Mac Spp1, Mac resident, Neutro Siglecf and Neutro Lcn2 in control and depleted groups; Mac : Macrophages, Mono/Mac Prolif: monocytes/macrophages proliferative, Neutro: Neutrophiles, Res_Mac: Resident Macrophages. Data are presented as individual, mean value +/- SEM. two-tailed Mann-Whitney test (d) and t test (e, f).

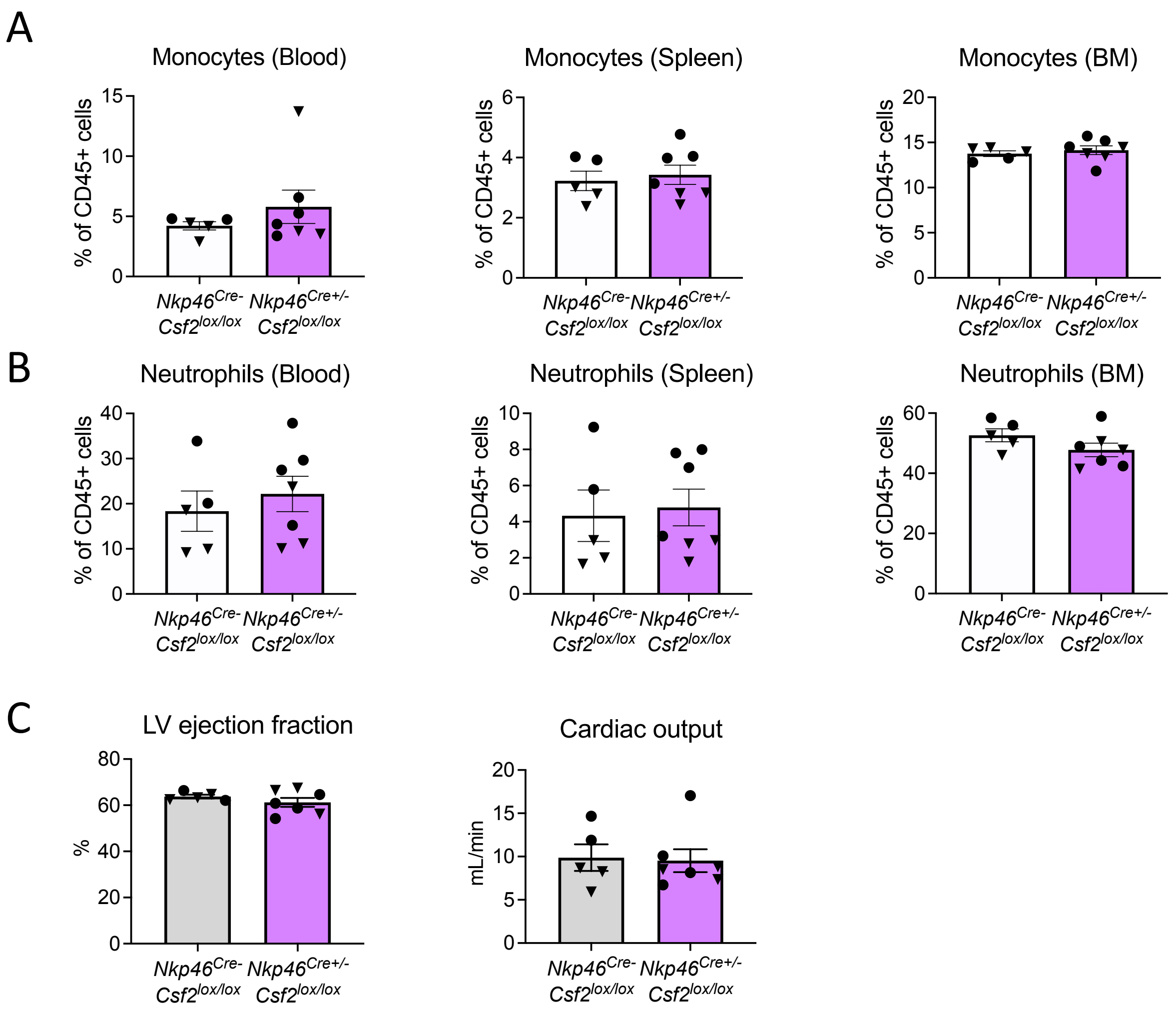

Supplementary Fig.20. baseline characterization of mice with specific deletion of GM-CSF in NK cells. A, proportions of CD11b+Ly6G-Ly6C+ monocytes in the blood, the spleen and the bone marrow in Nkp46Cre+/-Csf2lox/lox (n=5) and littermate Nkp46Cre-Csf2lox/lox control mice (n=8). B, proportions of CD11b+Ly6G+Ly6C- neutrophils in the blood, the spleen and the bone marrow in 8-week old Nkp46Cre+/-Csf2lox/lox (n=5) and littermate Nkp46Cre-Csf2lox/lox control mice (n=8). Circle for males and triangle for females animals. C, cardiac systolic function of mice evaluated using echocardiography. Circle for males and triangle for females animals. Data are presented as individual, mean value +/- SEM.

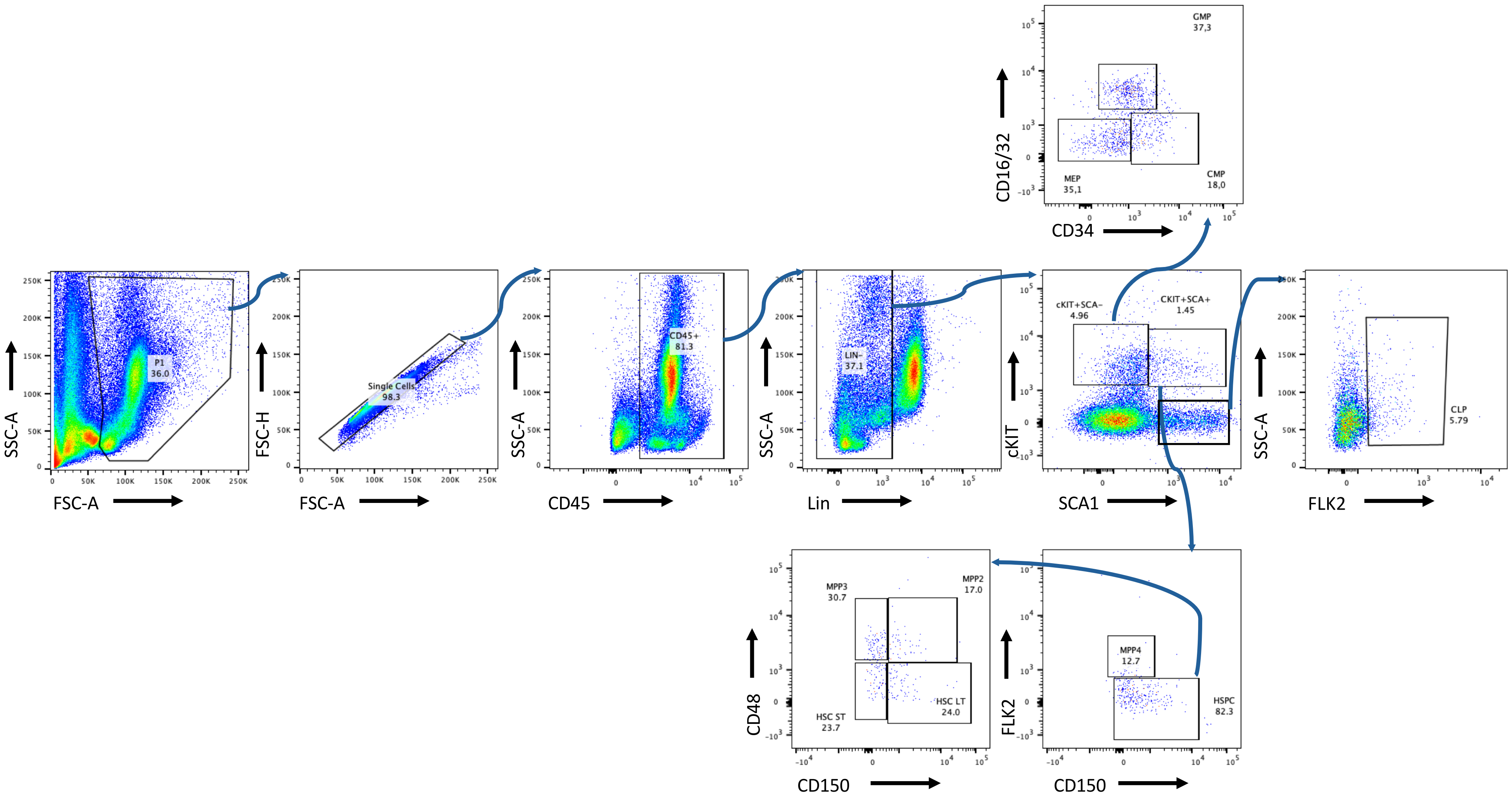

Supplementary Fig. 21. Gating strategy for bone marrow progenitors.

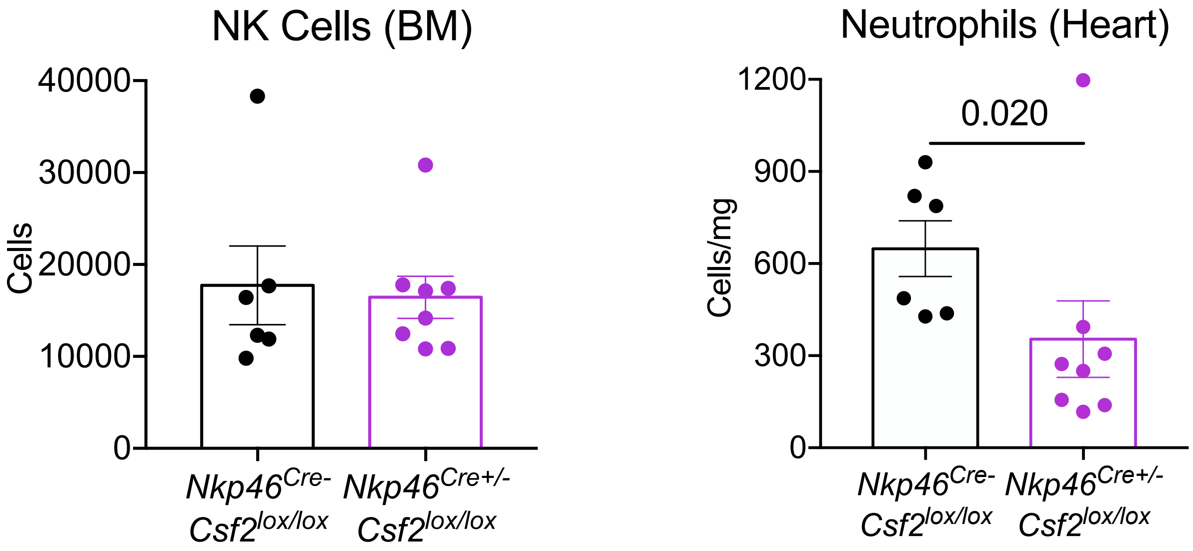

Supplementary Fig.22. GM-CSF deletion in NK cells. NK cell and neutrophil count in the BM at day 5 after MI in Nkp46Cre+/-Csf2lox/lox (n=8) and littermate Nkp46Cre-Csf2lox/lox control mice (n=6). Data are presented as individual, mean value +/-SEM. P values were calculated using two-tailed Mann-Whitney test.

	Controls (N=5)	Early MI (N=9)	Late MI (N=8)
Age (mean, years)	46	59	58
Male, n %	100%	100%	100%
Delay post MI	-	7 (2-13) days	39 (3 – 108) months
Hypertension	0/5	4/9	4/8
Dyslipidemia	0/5	9/9	7/8
Smokers	0/5	3/9	5/8
Obesity (BMI>30)	0/5	1/9	1/8
Diabetes	0/5	3/9	4/8
INTERMACS 1	0/5	9/9	1/8
INTERMACS 2-3	0/5	0/9	8/9
LVEF (%)	-	18.9	20.5

Supplementary Fig.23. Characteristic of patients included in the nanostring transcriptomic analysis. MI for myocardial infarction; BMI for Body Mass index; LVEF for left ventricule ejection fraction. INTERMACS classification (Stewart et al. Circ Heart Fail.2016)

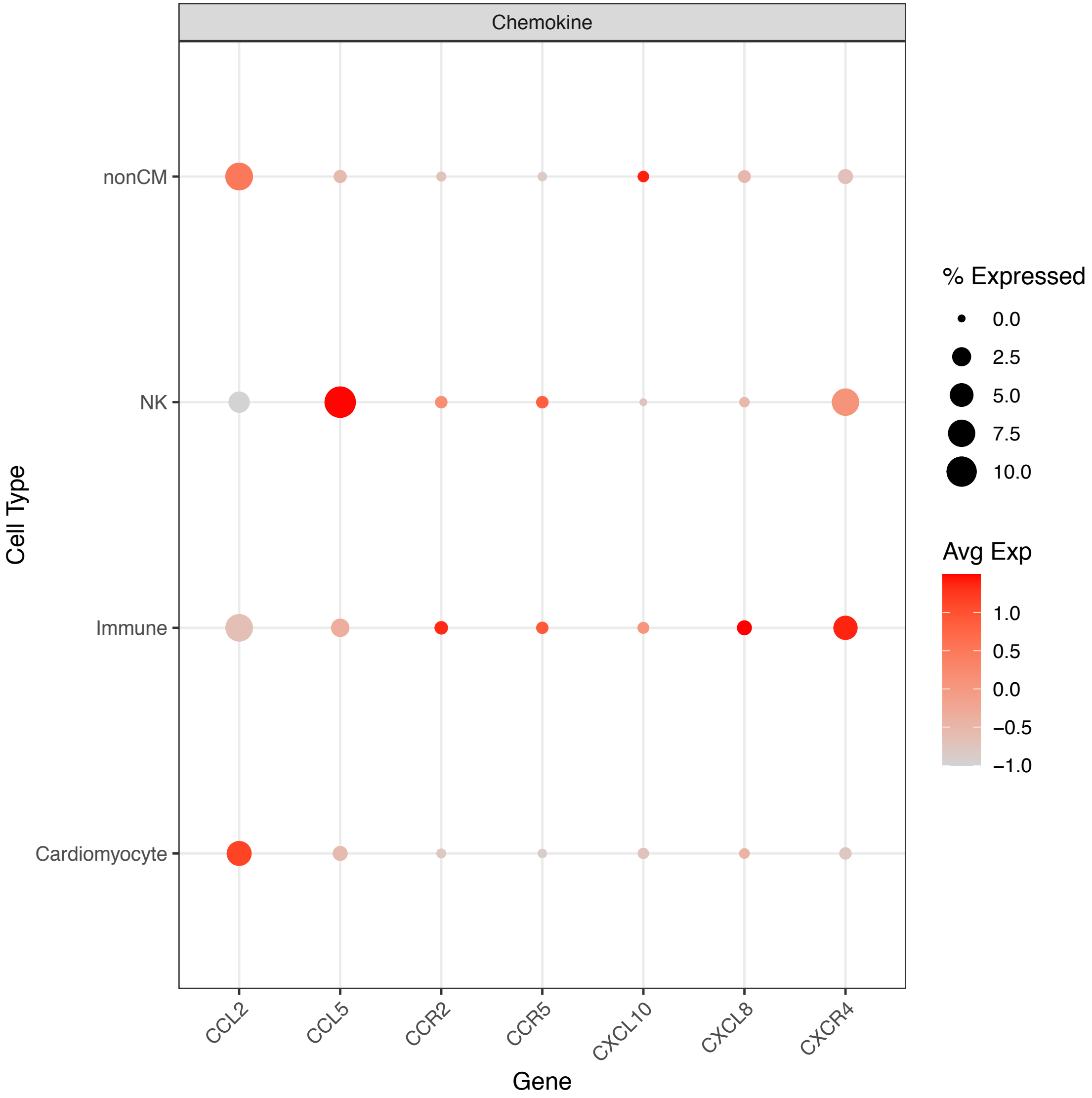

Supplementary Fig.24. Chemokines/chemokine receptors in human ischemic heart tissue. Dot plot representing average expression level of chemokine signaling genes in major cell types.

Primary Antibody	Fluorochrome	Clone	Supplier	Reference	Isotype	Stock. (mg/mL)	Dilution
**CCR2**	PE-Cy7	SA203G11	Biolegend	150612	Rat IgG2b, k	0,2	1 : 100
**CCR5**	BV421	C34-3448	BD Biosciences	743695	Rat IgG2c, k	0,2	1 : 100
**CD107a**	FITC	1D4B	BD Biosciences	553793	Rat IgG2a, k	0,5	1 : 100
**CD11b**	BV 605	M1/70	eBioscience	563015	Rat IgG2b, k	0,2	1 : 100
**CD11c**	alexa 700	N418	BioLegend	117320	Armenian Hamster IgG1, k	0,5	1 : 100
**CD150 (SLAM)**	BV711	TC15-12F12.2	BioLegend	115941	Rat IgG2a, k	0,2	1 : 100
**CD16/32**	FITC	93	Biolegend	101305	Rat IgG2b, k	0,5	1 : 100
**CD25**	PE-Cy7	PC61.5	eBioscience	25-0251-82	Rat IgG1, l	0,2	1 : 100
**CD3**	PerCP-Cy5,5	145-2C11	BD Biosciences	551163	Armenian Hamster IgG1, k	0,2	1 : 100
**CD335 - NKp46**	APC-eFluor780	29A1.4	eBioscience	47-3351-82	Rat IgG2a, k	0,2	1 : 100
**CD34**	PE-Cy7	MEC14.7	BioLegend	119325	Rat IgG2a, k	0,2	1 : 100
**CD4**	eFluor 450	RM4-5	eBioscience	48-0042-82	Rat IgG2a, k	0,2	1 : 100
**CD4**	FITC	RM4-5	eBioscience	11-0042-85	Rat IgG2a, k	0,5	1 : 100
**CD45**	PerCP	30-F11	BD Biosciences	557235	Rat IgG2b, k	0,2	1 : 100
**CD45**	BV510	30-F11	Biolegend	103137	Rat IgG2b, k	0,2	1 : 100
**CD45**	APC-eFluor 780	30-F11	eBioscience	47-0451-82	Rat IgG2b, k	0,2	1 : 100
**CD45R (B220)**	V500	RA3-6B2	BD Biosciences	561226	Rat IgG2a, k	0,2	1 : 100
**CD48**	PE	HM48-1	Biolegend	103405	Armenian Hamster IgG1, k	0,2	1 : 100
**CD64**	Brilliant Violet 421	X54-5/7.1	BioLegend	139309	Mouse IgG1, k	0,2	1 : 100
**CD8a**	Alexa Fluor 700	53-6.7	BD Biosciences	557959	Rat IgG2a, k	0,2	1 : 100
**cKIT / CD117**	APC-Cy7	2B8	Biolegend	105825	Rat IgG2b, k	0,2	1 : 100
**MHC II**	PerCP-eFluor 710	M5/114.15.2	eBioscience	46-5321-82	Rat IgG2b, k	0,2	1 : 100
**FLK2 / CD135**	APC	A2F10.1	BD Biosciences	560718	Rat IgG2a, k	0,2	1 : 100
**GMCSF**	PE	MP1-22E9	BD Biosciences	566245	Rat IgG2a, k	0,2	1 : 100
**Granzyme B**	Pacific Blue	gb11	BioLegend	515408	Mouse IgG1, k	0,2	1 : 100
**IFN-g**	FITC	XMG1.2	BD Biosciences	554411	Rat IgG1, k	0,5	1 : 100
**LIN**	BV421	-	BD Biosciences	561301	-		1 : 20
**Ly6C**	FITC	AL-21	BD Biosciences	553104	Rat IgM, k	0,5	1 : 100
**Ly6C**	APC-Cy7	AL-21	BD Biosciences	560596	Rat IgM, k	0,2	1 : 100
**Ly6C**	PE-Cy7	AL-21	BD Biosciences	560593	Rat IgM, k	0,2	1 : 100
**Ly6G**	PE	1A8	BD Biosciences	551461	Rat IgG2a, k	0,2	1 : 100
**NK1.1**	APC	PK136	eBioscience	17-5941-82	Mouse IgG2a, k	0,2	1 : 100
**SCA1**	BUV395	E13-161.7	BD Biosciences	744328	Rat IgG2a, k	0,2	1 : 100

Supplementary Table 1. General characteristics of antibodies used for flow cytometry characterization of immune cells.
