# Supplementary material for: NK cells promote cardiac cell death and regulate myelopoiesis in myocardial infarction
Source: Nat Commun. 2026 Apr 1;17:4699. doi: 10.1038/s41467-026-71334-x (PMC13212716; doi:10.1038/s41467-026-71334-x)
Supplement: Supplementary file 2 — Reporting Summary [file 41467_2026_71334_MOESM2_ESM.pdf]

nature portfolio

Corresponding author(s): Ait-Oufella

Last updated by author(s): Feb 13, 2026

Reporting Summary

Nature Portfolio wishes to improve the reproducibility of the work that we publish. This form provides structure for consistency and transparency in reporting. For further information on Nature Portfolio policies, see our Editorial Policies and the Editorial Policy Checklist.

Statistics

For all statistical analyses, confirm that the following items are present in the figure legend, table legend, main text, or Methods section.

n/a	Confirmed	
☐	☒	The exact sample size (*n*) for each experimental group/condition, given as a discrete number and unit of measurement
☐	☒	A statement on whether measurements were taken from distinct samples or whether the same sample was measured repeatedly
☐	☒	The statistical test(s) used AND whether they are one- or two-sided<br>*Only common tests should be described solely by name; describe more complex techniques in the Methods section.*
☐	☒	A description of all covariates tested
☐	☒	A description of any assumptions or corrections, such as tests of normality and adjustment for multiple comparisons
☐	☒	A full description of the statistical parameters including central tendency (e.g. means) or other basic estimates (e.g. regression coefficient) AND variation (e.g. standard deviation) or associated estimates of uncertainty (e.g. confidence intervals)
☐	☒	For null hypothesis testing, the test statistic (e.g. *F*, *t*, *r*) with confidence intervals, effect sizes, degrees of freedom and *P* value noted<br>*Give P values as exact values whenever suitable.*
☒	☐	For Bayesian analysis, information on the choice of priors and Markov chain Monte Carlo settings
☒	☐	For hierarchical and complex designs, identification of the appropriate level for tests and full reporting of outcomes
☐	☒	Estimates of effect sizes (e.g. Cohen's *d*, Pearson's *r*), indicating how they were calculated

Our web collection on statistics for biologists contains articles on many of the points above.

Software and code

Policy information about availability of computer code

Data collection	The authors declare that the data supporting the findings of this study are available within the paper and its supplementary information files. RNA-sequencing data of mouse heart generated for this report has been deposited in Gene Expression Omnibus (GSE317004). Transcriptomic data of human heart tissue generated using nanostring technology has been deposited in Gene Expression Omnibus (Released after acceptation) .<br>As described in the original work human data are available at cellxgene https://cellxgene.cziscience.com/collections/8191c283-0816-424b-9b61-c3e1d6258a77 and at the Zenodo data archive (https://zenodo.org/record/6578047). Raw data generated by CellRanger and SpaceRanger pipelines are available through the Human Cell Atlas Data Portal at https://data.humancellatlas.org/explore/projects/e9f36305-d857-44a3-93f0-df4e6007dc97 and at the Zenodo data archive (https://zenodo.org/record/6578553, https://zenodo.org/record/6578617 and https://zenodo.org/record/6580069).
Data analysis	Single-cell RNA-sequencing (Mouse)<br>Gene expression patterns in NK cells were analyzed in previously published single-cell RNA-seq data of total CD45+ cells from control and infarcted C57BL6/J mouse hearts (day 5 after MI) from reference (Rizzo et al Cardiovasc Res 2022). Briefly, cells corresponding to T and NK cells as identified in (Rizzo et al Cardiovasc Res 2022) were extracted and reclustered in Seurat v4 (Korsunsky et al. Nat Methods 2019), with batch correction of separate scRNA-seq libraries performed using harmony. Clustering was performed using 30 principal components and a 0.6 resolution. NK cells were identified based on the expression of surface NK1.1 as measured by cellular indexing of transcriptomes and epitopes by sequencing (CITE-seq) (Stoeckius et al. Nat Methods 2017).<br><br>Transcriptomic analysis (Nanostring)<br>RNA extraction. Up to three 20-µm thick sections were collected from FFPE tissue blocks to obtain 100 ng of RNA required for analyses. RNA was isolated and extracted using the tissue RNEasy FFPE kit #73504 (Qiagen, Hilden, Germany). The concentration and quality of isolated RNA

were assessed using NanoDrop 2000 spectrophotometer (Thermo Fisher Scientific Inc., Waltham, MA, USA).
RNA sequencing. Total RNA from each sample was hybridized with the nCounter® Human Banff Organ Transplant gene panel (B-HOT, NanoString Technologies, Seattle, WA, USA). This panel evaluates mRNA expression of 758 target genes and 12 selected housekeeping genes for data normalization. Internal quality reference for each assay was ensured by including a Panel Standard in each run: a pool of synthetic DNA oligonucleotides corresponding to the endogenous probes, allowing normalization of user, instrument, and lot-to-lot variations.
Quality control and data normalization. Raw counts of gene expression data were analyzed with the nSolver Analysis Software (version 4.0.70) and subjected to normalization using housekeeping genes. Background correction was applied, and the means of the supplied controls and housekeeping genes were used to normalize the measured expression values.
To evaluate the accuracy and quality of the gene expression input data, we applied quality control (QC) metrics (nSolver documentation) in all the samples included in the study. The QC assays refer to imaging, binding density, linearity of the positive controls, and limit of detection (LOD). If one or more of the four QC evaluations flagged an issue, the sample was removed from downstream analysis. Quantity and quality of isolated RNA were adequate in all cases and all the samples met the QC metrics.
All the samples passed the QC metrics which were defined as binding density > 0.05 and < 2.25 probes/µm2, % fields of view > 0.75, positive control linearity > 0.95, and sufficient assay efficiency. Per-sample LOD was defined as the mean of the counts of negative control probes for each sample and was ensured by using internal quality control reference probes. Raw gene expression data were normalized using the Remove Unwanted Variation (RUV) approach following quality control and housekeeping assessment.

Single-cell RNA-sequencing (Human)
As described in the original work, using single-nucleus RNA sequencing, we analyzed human heart data including thirty-one samples from twenty-three different individual hearts (Kuppe et al. Nature 2022). The samples were taken from the necrotic area (ischaemic zone from 12 samples), the unaffected LV myocardium regions (remote zone from 6 samples), patients with acute myocardial infarction, and the human heart specimen from the late stage of myocardial infarction (fibrotic zone from 6 samples). Four non-transplanted donor hearts were used as the control for these samples. These areas were identified based on discrete cardiac myocardial time points from the patients. There were 191 795 cells in all over 29 126 features in the data. We subtracted the data on natural killer cells because we were primarily interested in immune cells, and the result was 696 cells. Based on area, the data was further separated into 10, 30, and 100 days. We divided the patients into several time periods based on distinct zones of myocardial infarction in order to ascertain the function of NK cells in inflammation and cytotoxicity. 10, 30, and 100 days were the three distinct time periods into which the data was split comprising of 8, 1, and 2 patient samples, respectively. Next, we gathered the pathways linked to cytotoxicity or inflammation and used the AddModuleScore tool to get the scores for each cell. The scores for each pathway that displayed the gene expression trend linked with them were then obtained.

For manuscripts utilizing custom algorithms or software that are central to the research but not yet described in published literature, software must be made available to editors and reviewers. We strongly encourage code deposition in a community repository (e.g. GitHub). See the Nature Portfolio guidelines for submitting code & software for further information.

Data

Policy information about availability of data

All manuscripts must include a data availability statement. This statement should provide the following information, where applicable:
- Accession codes, unique identifiers, or web links for publicly available datasets
- A description of any restrictions on data availability
- For clinical datasets or third party data, please ensure that the statement adheres to our policy

The authors declare that the data supporting the findings of this study are available within the paper and its supplementary information files. RNA-sequencing data of mouse heart generated for this report has been deposited in Gene Expression Omnibus (Released after acceptation). Transcriptomic data of human heart tissue generated using nanostring technology has been deposited in Gene Expression Omnibus (Released after acceptation) .

Research involving human participants, their data, or biological material

Policy information about studies with human participants or human data. See also policy information about sex, gender (identity/presentation), and sexual orientation and race, ethnicity and racism.

Reporting on sex and gender	Extended data Fig.18
Reporting on race, ethnicity, or other socially relevant groupings	NA
Population characteristics	Extended data Fig.18
Recruitment	MI patients
Ethics oversight	Normal heart tissue was obtained from Creative Bioarray company (United States). The pathological human cardiac tissue samples were collected in the IRB-approved CVMR biobank in our institution (IRB-approval CVMR-PRB HEGP authorization CPP IDF2 2016-13-09 MS2). All the patients gave informed consents for the surgical procedure.<br>The protocol for human iPS was approved by the local institutional ethics committee (Comité de Protection des Personnes Ile de France XI IRB number 11-015). Human NK cells were isolated from blood donors (Collaboration Etablissement Français du sang) (IRB 2022-2026-046 Comité de Coordination de la Protection des Sujets et des Locaux Imagine). All the volunteers and blood donors gave informed consents.

Note that full information on the approval of the study protocol must also be provided in the manuscript.

Field-specific reporting

Please select the one below that is the best fit for your research. If you are not sure, read the appropriate sections before making your selection.

- [x] Life sciences
- [ ] Behavioural & social sciences
- [ ] Ecological, evolutionary & environmental sciences

For a reference copy of the document with all sections, see nature.com/documents/nr-reporting-summary-flat.pdf

Life sciences study design

All studies must disclose on these points even when the disclosure is negative.

Sample size	For ischemic cardic tissue characterization, sample size was determined based on the scientific litterature and our expertise. When possible, depending on the availability of the genetically-modified animals and colony production efficiency, a minimum of 10 mice were used per group to obtain statistically relevant cohorts.
Data exclusions	No exclusion
Replication	In vivo experiments performed in mice involving different mouse models to assess the role of NK cells in post-ischemic cardiac remodeling. Replication information is provided for each figure in the figure legends.
Randomization	No randomization
Blinding	The investigators were blinded to group allocation during data collection and analysis.

Reporting for specific materials, systems and methods

We require information from authors about some types of materials, experimental systems and methods used in many studies. Here, indicate whether each material, system or method listed is relevant to your study. If you are not sure if a list item applies to your research, read the appropriate section before selecting a response.

Materials & experimental systems

n/a	Involved in the study	
☐	☒	Antibodies
☒	☐	Eukaryotic cell lines
☒	☐	Palaeontology and archaeology
☐	☒	Animals and other organisms
☐	☒	Clinical data
☒	☐	Dual use research of concern
☒	☐	Plants

Methods

n/a	Involved in the study	
☒	☐	ChIP-seq
☐	☒	Flow cytometry
☒	☐	MRI-based neuroimaging

Antibodies

Antibodies used	Antibodies are described in supplemental table 1
Validation	The primary antibodies have been used according to the manufacturer's website instructions. The isotypes were used as negative controls.

Animals and other research organisms

Policy information about studies involving animals; ARRIVE guidelines recommended for reporting animal research, and Sex and Gender in Research

Laboratory animals	All mice used in this study were of the C57Bl/6J strain. Specifically, C57BL/6J mice (sourced from Janvier, France), Gzmb-/- mice (from Jackson Laboratory, USA), Nkp46iCre+/- R26RDTA mice (provided by E. Vivier's lab in Marseille, France), Ncr1gfp/gfp mice (from O. Mandelboim's lab in Jerusalem, Israel), and Csf2Lox/lox mice (from I.P. Wicks at WEHI in Melbourne, Australia) were used. Ccr2-/- mice were obtained from Jackson Laboratory (USA) and Apoe-/-Ccr5-/- (named Ccr5-/- in the manuscript) came from Y. Döring's Lab (Switzerland). All mice were aged 9-10 weeks at the time of myocardial infarction (MI) induction.
Wild animals	No wild animals were used in the study
Reporting on sex	All experiments have been done in males. Anti-NK1.1 experiments have been done in both males and females
Field-collected samples	no field-collected samples were used in the study

Ethics oversight	Experiments were conducted according to the guidelines formulated by the European Community for experimental animal use (L358-86/609EEC) and were approved by the Ethical Committee of INSERM and the French Ministry of Agriculture (agreement A75-15-32).

Note that full information on the approval of the study protocol must also be provided in the manuscript.

Clinical data

Policy information about clinical studies

All manuscripts should comply with the ICMJE guidelines for publication of clinical research and a completed CONSORT checklist must be included with all submissions.

Clinical trial registration	Normal heart tissue was obtained from Creative Bioarray company (United States). The pathological human cardiac tissue samples were collected in the IRB-approved CVMR biobank in our institution (IRB-approval CVMR-PRB HEGP authorization CPP IDF2 2016-13-09 MS2).
Study protocol	Patients with myocardial infarction
Data collection	age, gender, comorbidities, CV risk factors, LV ejection fraction
Outcomes	mortality

Plants

Seed stocks	*Report on the source of all seed stocks or other plant material used. If applicable, state the seed stock centre and catalogue number. If plant specimens were collected from the field, describe the collection location, date and sampling procedures.*
Novel plant genotypes	*Describe the methods by which all novel plant genotypes were produced. This includes those generated by transgenic approaches, gene editing, chemical/radiation-based mutagenesis and hybridization. For transgenic lines, describe the transformation method, the number of independent lines analyzed and the generation upon which experiments were performed. For gene-edited lines, describe the editor used, the endogenous sequence targeted for editing, the targeting guide RNA sequence (if applicable) and how the editor was applied.*
Authentication	*Describe any authentication procedures for each seed stock used or novel genotype generated. Describe any experiments used to assess the effect of a mutation and, where applicable, how potential secondary effects (e.g. second site T-DNA insertions, mosiacism, off-target gene editing) were examined.*

Flow Cytometry

Plots

Confirm that:

- [x] The axis labels state the marker and fluorochrome used (e.g. CD4-FITC).
- [x] The axis scales are clearly visible. Include numbers along axes only for bottom left plot of group (a 'group' is an analysis of identical markers).
- [x] All plots are contour plots with outliers or pseudocolor plots.
- [x] A numerical value for number of cells or percentage (with statistics) is provided.

Methodology

Sample preparation	Blood, bone marrow, spleen, heart tissue samples were collected at sacrifice for analysis of leukocyte subsets.
Instrument	Cells were acquired using a BD LSRII Fortessa flow cytometer (BD Biosciences)
Software	Cells were analyzed with FlowJo (Becton Dickinson & Company (BD) version 10.9.0
Cell population abundance	Minimum of 0.5 million of cells were analyzed in each flow cytometry sample
Gating strategy	Classical monocytes were defined as NK1.1-CD11b+Ly6G-Ly6Chigh cells. Non-classical monocytes were defined as NK1.1-CD11b+Ly6G-Ly6Clow cells. Neutrophils were defined as NK1.1-CD11b+Ly6G+ cells. CD4+ T Lymphocytes were selected as NK1.1-B220-CD11b-CD3+CD4+ cells. CD8+ T Lymphocytes were selected as NK1.1-B220-CD11b- CD3+CD8+ cells. B cells were defined as NK1.1-CD11b-B220+ cells. NK cells were defined as CD11b-CD3-NK1.1+Nkp46+

- [x] Tick this box to confirm that a figure exemplifying the gating strategy is provided in the Supplementary Information.
